# Clinical features and mortality risk factors in non-HIV elderly patients with cryptococcal meningitis: A retrospective cohort study from 2013 to 2022

**DOI:** 10.1371/journal.pntd.0013521

**Published:** 2025-09-11

**Authors:** Xiaofeng Xu, Xiaohong Su, Weipeng Li, Li Xu, Dongcheng Li, Kai Dai, Junyu Liu, Jia Liu, Fuhua Peng, Ying Jiang

**Affiliations:** 1 Department of Neurology, Third Affiliated Hospital of Sun Yat-Sen University, Guangzhou, Guangdong Province, China; 2 School of Traditional Chinese Medicine, Southern Medical University, Guangzhou, Guangdong Province, China; Albert Einstein College of Medicine, UNITED STATES OF AMERICA

## Abstract

**Background:**

Cryptococcal meningitis (CM) is a life-threatening fungal infection with increasing incidence among non-HIV (human immunodeficiency virus) elderly populations. However, data on CM in non-HIV elderly patients are limited. This study aimed to analyze the clinical features, outcomes, and prognostic factors in non-HIV elderly CM patients using the largest dataset to date.

**Methods:**

A retrospective cohort study was conducted using data from 667 non-HIV CM patients treated between 2013 and 2022. Patients were categorized into elderly (≥60 years) and non-elderly groups. Clinical features, laboratory findings, and neuroimaging results were analyzed. Least Absolute Shrinkage and Selection Operator regression identified prognostic factors, and multivariate logistic regression was used to construct a nomogram for predicting mortality. The model’s discrimination, calibration, and decision curve analysis (DCA) were evaluated.

**Results:**

Elderly patients accounted for 23.5% of the study population, exhibited distinct clinical characteristics, and had a significantly higher one-year all-cause mortality rate (31.2% [95% confidence interval (CI) 23.61-38.71] *vs*. 13.8% [95% CI 10.77-16.81], *P* < 0.001). Four prognostic factors for elderly patients were identified, and a predictive nomogram was developed. The predictive model achieved an area under the curve (AUC) of 0.81 (95% CI 0.71-0.91), and the AUC was 0.79 (95% CI 0.70-0.87) in the internal validation. The model was well-calibrated, and DCA indicated a net benefit.

**Conclusion:**

Non-HIV elderly CM patients present distinct clinical characteristics and have a higher mortality risk. The predictive model may facilitate the early identification of high-risk patients and guide timely interventions.

## Authors’ summary

Cryptococcal meningitis (CM) is a life-threatening fungal infection increasingly recognized in non-HIV (human immunodeficiency virus) elderly populations, yet its clinical characteristics and prognostic factors remain poorly understood. In this retrospective cohort study of 667 non-HIV CM patients (2013–2022), we identified distinct clinical features in elderly patients (≥60 years), who accounted for 23.5% of cases. Compared to non-elderly patients, elderly individuals exhibited higher rates of altered consciousness (23.6% *vs*. 12.2%) and functional disability, but fewer typical symptoms like headache (84.7% *vs*. 92.2%). Notably, one-year mortality was significantly elevated in elderly patients (31.2% *vs.* 13.8%). We developed the first nomogram incorporating four key predictors (modified Rankin Scale score, *Cryptococcus* smear count, leukocyte count, and basal ganglia lesions) to stratify mortality risk in this population, with an AUC of 0.81. The model demonstrated robust calibration and clinical utility in decision curve analysis. Our findings highlight the urgent need for age-specific diagnostic and management strategies. The proposed nomogram may aid clinicians in early identification of high-risk elderly patients, enabling timely interventions to improve outcomes. This study provides the largest dataset to date on non-HIV elderly CM patients, addressing a critical gap in the understanding of this vulnerable population.

## Introduction

Fungal pathogens affect over a billion individuals globally, with invasive fungal infections resulting in a mortality rate that surpasses those of higher-profile diseases such as tuberculosis and malaria, causing more deaths annually [1]. Among these, cryptococcal meningitis (CM) is the most life-threatening fungal disease, with an annual toll of over 181,000 deaths worldwide. Untreated cases have a mortality rate approaching 100% [2].

CM is traditionally linked to immunosuppressed patients, especially those with the human immunodeficiency virus (HIV). However, recent years have seen a rise in cases among non-HIV elderly populations, particularly in regions with aging demographics [3,4]. Elderly CM patients face unique challenges, displaying subtler, atypical symptoms but suffering from higher mortality rates [5]. Age-related immune decline and comorbidities such as diabetes, chronic kidney disease, and malignancy complicate diagnosis and treatment, highlighting the need for targeted research on this subgroup.

Despite growing awareness, data on non-HIV elderly CM patients remain sparse, with most studies involving small sample sizes [6,7].This study aims to address this gap by analyzing 667 non-HIV CM cases, including 157 elderly patients (≥60 years), representing the largest dataset to date. The analysis focuses on clinical features, outcomes, and mortality risk factors specific to elderly patients.

By leveraging this comprehensive dataset, our research will enhance the understanding of CM in the non-HIV elderly population. We also developed prognostic models to predict poor outcomes, offering crucial insights for optimizing treatment strategies and improving prognosis. These insights may lead to improvements in our clinical practice to better meet the needs of non-HIV elderly individuals, potentially reducing the morbidity and mortality.

## Methods

### Ethics statement

Ethical approval was granted by the Institutional Review Board (IRB) of the Third Affiliated Hospital of Sun Yat-sen University (ethics No. [2021]02-264-01). Due to the retrospective nature of this study and anonymous analysis of patient records, the exemption of informed consent was approved.

### Study population

**Fig 1:**
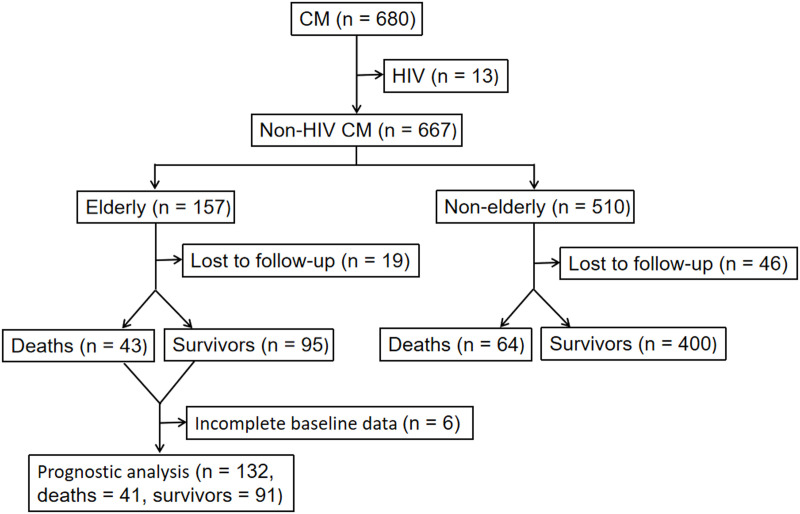
Flow chart of patient selection. A total of 667 non-HIV patients with CM were enrolled. Among them, 157 were elderly (≥60 years). In the elderly group, 19 patients were lost to follow-up and 6 had incomplete data, leaving 132 patients eligible for prognostic analysis (41 deaths and 91 survivors). CM = cryptococcal meningitis; HIV = human immunodeficiency virus.

This retrospective cohort study included non-HIV CM patients treated between January 1, 2013, and December 31, 2022, at the Third Affiliated Hospital of Sun Yat-sen University, which is a tertiary general hospital with approximately 2000 ward beds and the major clinical institution focusing on HIV-negative cryptococcosis in China. Inclusion and exclusion criteria: CM patients were identified through our hospital’s electronic medical record (EMR) system and confirmed by microbiological testing, with at least one of the following positive findings: cerebrospinal fluid (CSF) India ink stain, CSF *Cryptococcus* culture, CSF cryptococcal antigen (CrAg) test, or histopathological evidence of encapsulated yeasts (5–10 μm) in the meninges or brain [8]. Patients who tested positive for HIV antibodies were excluded. Patients aged ≥60 years were classified as elderly, while others were categorized as non-elderly. The selection flow diagram is presented in Fig 1.

### Data collection

Demographic characteristics, comorbidities, risk factors, clinical symptoms, illness duration, clinical assessments, laboratory results, neuroimaging findings (assessed by magnetic resonance imaging [MRI]), and lumbar puncture parameters were extracted from the EMR, with data collected within the first week of hospitalization defined as baseline.

Antifungal treatment regimens were extracted from EMR. Follow-up information was obtained from inpatient and outpatient records and supplemented by the telephone follow-up, which was conducted by the research team at least every three months. Outpatient visits and telephone follow-up focused on medication use, living status, and survival outcomes, while inpatient follow-up included additional laboratory tests, microbiological assessments, and imaging evaluations. One-year all-cause mortality and the timing of death were documented, and follow-up duration was defined as the interval from diagnosis to the last follow-up or death. One-year all-cause mortality was subsequently analyzed.

### Definitions

*Cryptococcus* smear counting in CSF was performed using India ink staining as follows: 1) CSF samples were collected into sterile tubes, the volume was recorded, and the samples were centrifuged at 3000 rpm for 10 minutes; 2) the sediment was smeared onto a glass slide and mixed evenly with India ink at a 10:1 ratio; 3) a coverslip was placed over the mixture, which was left to stand for 2–3 minutes; yeast cells were then identified under low-power microscopy and counted under high-power magnification. The Glasgow Coma Scale (GCS) and the modified Rankin Scale (mRS) scores were obtained from the EMR. The GCS is a standardized tool for assessing consciousness, with a total score ranging from 3 to 15 based on eye opening (1–4), verbal response (1–5), and motor response (1–6); lower scores indicate more severe impairment. In this study, altered consciousness was defined as a GCS score < 15. The mRS is a widely used measure of functional disability, with scores ranging from 0 to 6: 0 = no symptoms; 1 = no significant disability despite symptoms; 2 = slight disability; 3 = moderate disability; 4 = moderately severe disability; 5 = severe disability; and 6 = death. Onset time referred to symptom appearance prompting medical attention, while diagnosis date was defined by the first sample confirming *Cryptococcus*. Rural/urban classification was based on patient residency during symptom onset.

### Statistical analysis

To describe demographic characteristics, frequencies and proportions were reported for categorical variables, while means with standard errors or medians with interquartile ranges (IQRs) were used for continuous variables. Categorical variables were analyzed using the chi-square or Fisher’s exact test. Continuous variables were assessed with Student’s t-test or Mann-Whitney U test, depending on data distribution. Kaplan-Meier curves and log-rank tests were used to analyze survival distributions.

During the model development stage, univariate analysis was conducted using the chi-square test and Student’s t-test to identify differences between survivors and death. When appropriate, the Fisher’s exact test and the Mann-Whitney U test were used. The discriminatory capacity of variables in univariate analyses was determined by calculating the area under the curve (AUC). The strength and direction of the linear relationships between continuous variables were assessed using the of the R package “corrplot”. A 10-fold cross-validated Least Absolute Shrinkage and Selection Operator (LASSO) regression analysis was employed to reduce data dimensionality and key predictors. The nomogram was developed by incorporating these predictors based on their regression coefficients in the multivariate logistic regression model [9]. The discrimination of the nomogram was assessed using the area under the receiver operating characteristic (ROC) curve and concordance index (C-index), with sensitivity analysis performed through the ROC. Calibration was evaluated using calibration curves and Hosmer-Lemeshow tests (H-L test). Both discrimination and calibration were further validated using bootstrapping methods with 1,000 resamples [10]. Additionally, decision curve analysis (DCA) was employed to assess the net benefit of the model for patients [11].

Statistical analyses were performed with IBM SPSS (v27.0.1) and R (v4.4.0), with *P* < 0.05 considered significant.

## Results

### Baseline patient characteristics

**Fig 2:**
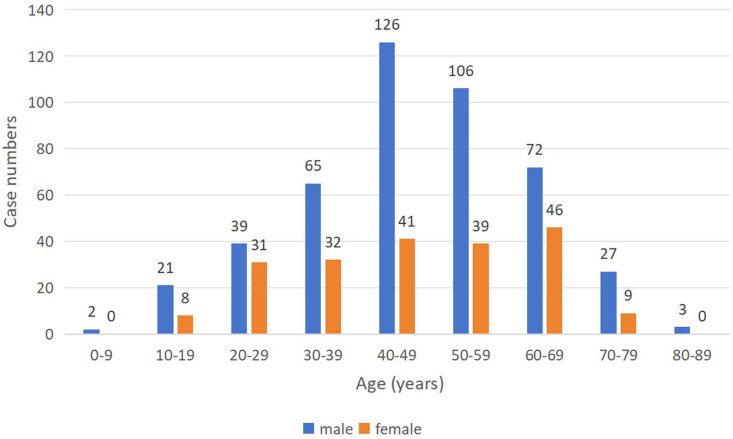
The age and gender distribution of the 667 patients with cryptococcal meningitis. The bar graph shows the number of cases (y-axis) stratified by age groups (x-axis) and gender (male: blue; female: red). Elderly patients (≥60 years) comprised 23.5% (157/667) of the cohort.

This study included 667 non-HIV CM patients (age range 5–83 years), comprising 461 males and 206 females. Age and gender distribution are shown in Fig 2. Elderly patients (≥60 years) accounted for 23.5% (157/667), while non-elderly patients made up 76.5% (510/667). Baseline characteristics are presented in Table 1.

**Table 1:** Baseline characteristics of elderly and non-elderly patients with cryptococcal meningitis.

Parameters	Elderly group (n = 157)	Non-elderly group (n = 510)	*P*-value
**Age (years)**	65 (62.5–69)	43 (32–51)	–
**Gender, male/female**	102/55	358/152	0.237
**Area type, urban/rural**	70/87	234/276	0.784
**Predisposing condition, n (%)**	53 (33.8)	133 (26.1)	0.067
** Diabetes mellitus, n (%)**	32 (20.4)	44 (8.6)	< 0.001
** Malignancy, n (%)**	8 (5.1)	13 (2.6)	0.120
** Organ transplant, n (%)**	2 (1.3)	8 (1.6)	1.000
** Systemic lupus erythematosus, n (%)**	4 (2.6)	29 (5.7)	0.141
**Immunological kidney disease, n (%)**	5 (3.2)	30 (5.9)	0.223
** Rheumatoid Arthritis, n (%)**	3 (1.9)	11 (2.2)	1.000
**Others, n (%)**	12 (7.6)	18 (3.5)	0.045
**Fever, n (%)**	71 (45.2)	225 (44.1)	0.854
**Headache, n (%)**	133 (84.7)	470 (92.2)	0.008
**Vomiting, n (%)**	63 (40.1)	257 (50.4)	0.028
**Reduced vision, n (%)**	29 (18.7)	127 (24.9)	0.106
**Reduced hearing, n (%)**	16 (10.2)	50 (9.8)	0.879
**Onset to diagnosis time (days), median (IQR)**	26 (15–38)	22 (13–44)	0.321
**Glasgow coma scale < 15, n (%)**	37 (23.6)	62 (12.2)	0.001
**Modified Rankin scale, median (IQR)**	1 (1–4)	1 (1–2)	0.009
**Alanine aminotransferase (U/L), median (IQR)**	23 (14–35.5)	27 (16–45)	0.008
**Aspartate aminotransferase (U/L), median (IQR)**	19 (15–26)	21 (14–31)	0.136
**Serum creatinine (umol/L), median (IQR)**	67 (50–86)	65 (54–81)	0.772
**Serum urea (umol/L), median (IQR)**	200.0 (151.7–310)	250.0 (169.8–346.6)	0.005
**Total leukocyte count (10** ^**9**^ **/L), median (IQR)**	8.90 (6.4–11.7)	8.740 (6.4–11.9)	0.346
**Hemoglobin (g/dl), median (IQR)**	124 (112.5–137)	125 (109–140)	0.568
**Platelet count (10** ^**9**^ **/L), median (IQR)**	235.00 (181–293)	254 (199.5–310.5)	0.008
**Lymphocyte count (10** ^**9**^ **/L), median (IQR)**	1.00 (0.7–1.5)	1.210 (0.8–1.8)	0.002
**Neutrophil count (10** ^**9**^ **/L), median (IQR)**	6.7 (4.7–9.6)	6.7 (4.6–9.7)	0.588
**Monocyte count (10** ^**9**^ **/L), median (IQR)**	0.6 (0.4–0.8)	0.6 (0.4–0.8)	0.732
**Serum albumin (g/L), median (IQR)**	38.2 (34.4–41.2)	39.5 (35.9–43.1)	0.002
**Serum globulin (g/L), median (IQR)**	25.3 (22.7–29.1)	25.9 (23.1–29.2)	0.435
**Lumbar puncture**	**Elderly group (n = 150)**	**Non-elderly group (n = 485)**	
**Open pressure**			
** > 300 mmH**_**2**_ **O, n (%)**	47 (31.3)	227 (46.8)	0.001
** > 200, ≤ 300 mmH**_**2**_ **O, n (%)**	42 (28.0)	117 (24.1)	0.395
** ≤ 200 mmH**_**2**_ **O, n (%)**	61 (40.7)	141 (29.1)	0.010
**Total cell count (per ml), median (IQR)**	87 (40–176)	87 (32.5-176)	0.62
**Protein (g/L), median (IQR)**	0.9 (0.6–1.6)	0.7 (0.5-1.2)	< 0.001
**Elevated protein** ^**a**^ **, n (%)**	135 (90.0)	369 (76.1)	< 0.001
**Glucose (mmol/L), median (IQR)**	1.6 (0.7–2.6)	1.8 (0.9-2.7)	0.203
**Hypoglycorrhachia** ^**b**^ **, n (%)**	108 (72.0)	337 (69.5)	0.543
**Chloride (mmol/L), mean ± SD**	115.1 ± 7.4	117.8 ± 7.4	< 0.001
**Hypochloremia** ^**c**^	124 (82.7)	330 (68.0)	< 0.001
***Cryptococcus* smear count (/L), median (IQR)**	860 (0–11448)	516 (0-8311)	0.593
**India Ink Positives, n (%)**	129 (86.0)	451 (93.0)	0.013
**Culture Positives, n (%)**	108 (72.0)	316 (65.2)	0.145
***Cryptococcus* classification (*neoformans/gattii*)** ^**d**^	64/1	178/16	0.080
**Neuroimaging findings**	**Elderly group (n = 151)**	**Non-elderly group (n = 487)**	
**Meningeal enhancement, n (%)**	83 (54.9)	224 (46.0)	0.062
**Hydrocephalus, n (%)**	39 (25.8)	98 (20.1)	0.141
**Acute infarction, n (%)**	15 (9.9)	8 (1.6)	< 0.001
**Basal ganglia lesions, n (%)**	40 (26.5)	192 (39.4)	0.004
**Cryptococcoma, n (%)**	3 (2.0)	12 (2.5)	1.000
**Mortality rate, % (deaths/total)**	31.2 (43/138)	13.8 (64/464)	< 0.001

Others refers to predisposing conditions for cryptococcal meningitis other than those specifically listed in the table.

^a^Cerebrospinal fluid protein level ≥ 0.45 g/L.

^b^Cerebrospinal fluid glucose level ≤ 2.5 mmol/L or cerebrospinal fluid: blood glucose ratio 0.5.

^c^Cerebrospinal fluid chloride level ≤ 121 mmol/L.

^d^*Cryptococcus* can be classified based on culture results since October 2017.

IQR = interquartile range; SD = standard deviation.

Approximately one-third of both groups had predisposing conditions for CM, with diabetes significantly more common in elderly patients (20.4% *vs*. 8.6%, *P* < 0.001). Common symptoms included fever, headache, and vomiting, though headache and vomiting were less frequent in elderly patients (headache: 84.7% *vs*. 92.2%, *P* = 0.008; vomiting: 40.1% *vs*. 50.4%, *P* = 0.028). Elderly patients exhibited significantly higher rates of altered consciousness (GCS < 15: 23.6% *vs*. 12.2%, *P* = 0.001) and greater functional disability (mRS score: 1 [IQR 1–4] *vs*. 1 [IQR 1–2], *P* = 0.009).

Elderly patients were less likely to have lumbar puncture opening pressures >300 mmH_2_O (31.3% *vs*. 46.8%, *P* = 0.001). CSF analysis revealed higher protein levels and lower chloride levels in elderly patients (protein: 0.9 [IQR 0.6-1.6] g/L *vs*. 0.7 [IQR 0.5-1.2] g/L, *P* < 0.001; chloride: 115.1 ± 7.4 mmol/L *vs*. 117.8 ± 7.4 mmol/L, *P* < 0.001). Despite a lower positive India ink smear rate, initial *Cryptococcus* counts did not differ significantly between groups (860 [IQR 0–11448]/L *vs*. 516 [IQR 0–8311]/L, *P* = 0.593).

**Fig 3:**
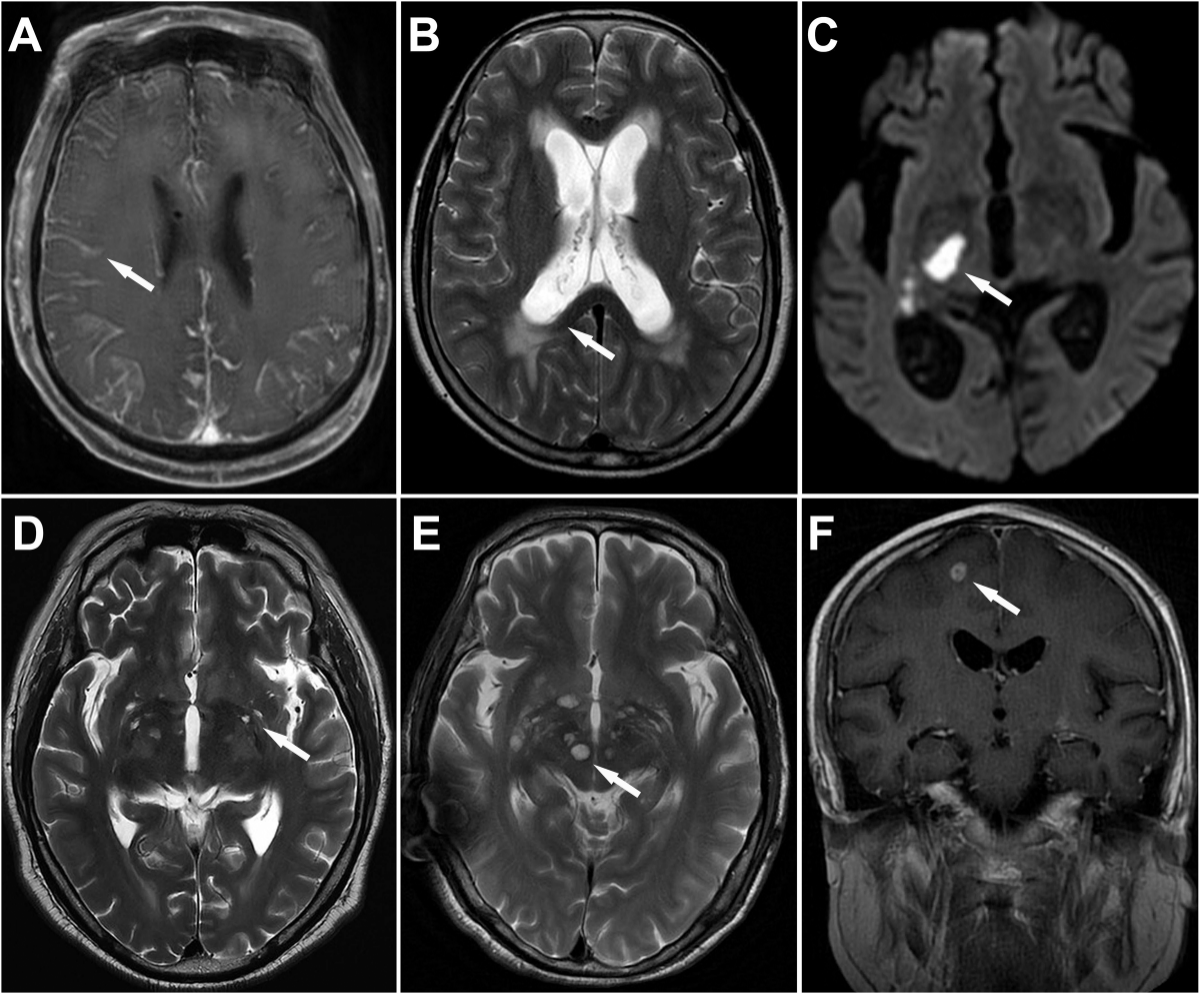
Representative MRI findings in elderly patients with cryptococcal meningitis. (A) Gadolinium contrast-enhanced T1WI shows meningeal enhancement (arrows). (B) T2WI shows hydrocephalus (arrows). (C) DWI shows a acute cerebral infarct (arrows). (D) T2W1 shows basal ganglia lesions of Virchow-Robin spaces dilatation (arrows). (E) T2W1 shows basal ganglia lesions of gelatinous pseudocysts (arrows). (F) Gadolinium contrast-enhanced T1WI shows cryptococcoma (arrows). DWI = Diffusion-weighted image; MRI = magnetic resonance imaging; T1WI = T1-weighted image; T2WI = T2-weighted image.

Imaging studies showed that meningeal enhancement was the most common finding (~50%) without significant differences between groups. However, acute cerebral infarction was more prevalent among elderly patients (9.9% *vs*. 1.6%, *P* < 0.001). Basal ganglia lesions, including Virchow-Robin (VR) spaces dilatation and gelatinous pseudocysts, were less frequent in elderly patients (26.5% *vs*. 39.4%, *P* = 0.004). The typical imaging findings in elderly patients are shown in Fig 3.

**Fig 4:**
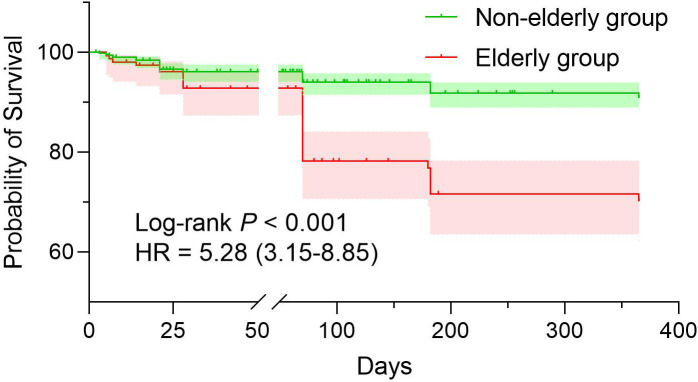
Kaplan-Meier survival analysis comparing elderly (≥60 years) and non-elderly (<60 years) cryptococcal meningitis patients. The survival probability (y-axis) over time (x-axis, days) is significantly lower in elderly patients (red curve) versus non-elderly patients (blue curve) (log-rank *P* < 0.001; hazard ratio [HR] = 5.28, 95% confidence interval 3.15-8.85). One-year mortality rates were 31.2% (elderly) and 13.8% (non-elderly).

All patients primarily received induction therapy regimens based on amphotericin B and triazole antifungals. A total of 138 elderly and 464 non-elderly patients completed the one-year follow-up (90.3%), with 19 and 46 patients lost to follow-up, respectively. The one-year all-cause mortality were significantly higher in elderly patients (31.2% [95% confidence interval (CI) 23.61-38.71] vs. 13.8% [95% CI 10.77-16.81], *P* < 0.001). Kaplan-Meier analysis confirmed worse survival outcomes for elderly patients (hazard ratio 5.28, [95% CI 3.15-8.85], *P* < 0.001) (Fig 4).

### Mortality risk factors in elderly patients

After excluding 25 cases with incomplete baseline data or loss to follow-up, 132 elderly patients were included in prognostic analysis. Surviving and deceased patients’ demographic and clinical features are summarized in Table 2. Univariate analysis indicated that deceased patients had higher rates of GCS < 15, opening pressure >300 mmH_2_O, basal ganglia lesions, elevated mRS scores, total leukocyte count, neutrophil count, and initial *Cryptococcus* smear count.

**Table 2:** Baseline characteristics predicting mortality in elderly patients with cryptococcal meningitis.

Parameters	Survivors (n = 91)	Deaths (n = 41)	*P*-value
**Age (years), median (IQR)**	65 (61–69)	67 (63.5–70)	0.099
**Gender, male/female**	61/30	30/11	0.616
**Immunodeficiency, n (%)**	28 (30.8)	17 (41.5)	0.317
**Headache, n (%)**	79 (86.8)	31 (75.6)	0.178
**Fever, n (%)**	39 (42.9)	25 (61.0)	0.082
**Vomiting, n (%)**	37 (40.7)	16 (39.0)	0.988
**Reduced vision, n (%)**	14 (15.4)	8 (19.5)	0.864
**Reduced hearing, n (%)**	7 (7.7)	7 (17.1)	0.189
**Onset to diagnosis time (days), median (IQR)**	23 (14-37)	28 (14.5–38.5)	0.762
**Glasgow coma scale < 15, n (%)**	15 (16.5)	19 (46.3)	0.001
**Modified Rankin scale, median (IQR)**	1 (1–3)	4 (1–5)	< 0.001
**Alanine aminotransferase (U/L), median (IQR)**	19 (15–25)	19 (15.5–27)	0.412
**Aspartate aminotransferase (U/L), median (IQR)**	22 (13–35)	30 (16–42.5)	0.150
**Serum creatinine (umol/L), median (IQR)**	69 (63–83)	69 (45–95.5)	0.920
**Serum urea (umol/L), median (IQR)**	200 (152-309)	219.1 (150.9–349)	0.577
**Total leukocyte count (10** ^**9**^ **/L), median (IQR)**	7.9 (6.0–11.0)	10.4 (6.9–14.8)	0.006
**Hemoglobin (g/dl), mean ± SD**	123.0 ± 19.3	124.9 ± 21.8	0.622
**Platelet count (10** ^**9**^ **/L), median (IQR)**	232.0 (184–300)	235 (182–287)	0.877
**Urban/Rural**	37/54	22/19	0.230
**Lymphocyte count (10** ^**9**^ **/L), median (IQR)**	1.0 (0.7–1.5)	0.9 (0.6–1.4)	0.270
**Neutrophil count (10** ^**9**^ **/L), median (IQR)**	6.3 (4.6–8.9)	8.4 (5.2–12.8)	0.006
**Monocyte count (10** ^**9**^ **/L), median (IQR)**	0.6 (0.4-0.8)	0.6 (0.4-0.9)	0.857
**Serum albumin (g/L), mean ± SD**	38.9 ± 4.7	36.6 ± 5.5	0.107
**Serum globulin (g/L), median (IQR)**	26.3 (23.2–30.1)	25.2 (22.6–29.1)	0.336
**Open pressure**			
** > 300 mmH**_**2**_ **O, n (%)**	21 (23.1)	18 (43.9)	0.026
** > 200, ≤ 300 mmH**_**2**_ **O, n (%)**	28 (30.8)	10 (24.4)	0.588
** ≤ 200 mmH**_**2**_ **O, n (%)**	42 (46.2)	13 (31.7)	0.172
**Total cell count (per ml), median (IQR)**	96 (42–184)	72 (30-163)	0.299
**Protein (g/L), median (IQR)**	1.0 (0.7–1.9)	0.8 (0.6-1.4)	0.139
**Glucose (mmol/L), mean ± SD**	1.9 ± 1.3	1.6 ± 1.2	0.222
**Chloride (mmol/L), mean ± SD**	114.7 ± 7.0	114.7 ± 8.7	0.998
***Cryptococcus* smear count (/L), median (IQR)**	178.0 (0-3989)	4704 (73-40950)	0.001
**India Ink Positives, n (%)**	72 (79.1)	38 (92.7)	0.093
**Culture Positives, n (%)**	64 (70.3)	26 (63.4)	0.557
**Meningeal enhancement, n (%)**	54 (59.3)	22 (53.7)	0.674
**Hydrocephalus, n (%)**	24 (26.4)	11 (26.8)	0.874
**Acute infarction, n (%)**	9 (9.9)	6 (14.6)	0.618
**Basal ganglia lesions**	17 (18.7)	20 (48.8)	< 0.001
**Cryptococcoma, n (%)**	2 (2.2)	0 (0.0)	0.852

IQR = interquartile range; SD = standard deviation.

To identify key predictors, AUC values and correlations among variables were calculated (S1 Fig). LASSO regression with 10-fold cross-validation was employed, and the optimized hyperparameter λ was determined based on the bivariate deviation (S2 Fig). Four variables were selected as the most valuable predictors for mortality in elderly patients, including: mRS scores, initial *Cryptococcus* smear count, total leukocyte count, and the presence of basal ganglia lesions.

### Establishment of the nomogram

**Fig 5:**
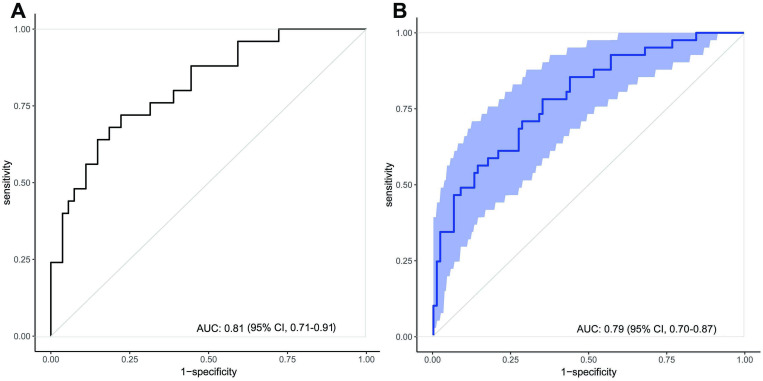
ROC curves for the mortality prediction model. (A) ROC curve of the nomogram in the derivation cohort (AUC = 0.81, 95% confidence interval: 0.71-0.91). (B) Internal validation via bootstrap resampling (n = 1,000) confirmed model stability (AUC = 0.79, 95% confidence interval 0.70-0.87). The light blue shaded area represents the 95% confidence interval. AUC = area under the curve; ROC = receiver operating characteristic.

**Fig 6:**
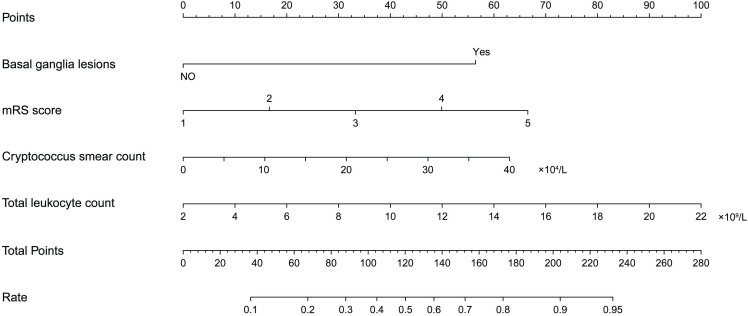
Nomogram for predicting one-year all-cause mortality in elderly patients with cryptococcal meningitis. The nomogram integrates four variables: basal ganglia lesions (present or absent), mRS score at admission (1-5), *Cryptococcus* smear count in CSF (×10^4^/L), and total peripheral blood leukocyte count (×10^9^/L). To use the nomogram, locate the patient’s value on each variable axis and draw a vertical line to the “Points” row to determine the score for each predictor. Sum the scores to obtain the “Total Points”, then locate this total on the corresponding axis to determine the predicted probability (Rate) of one-year all-cause mortality. CSF = cerebrospinal fluid; mRS = Modified Rankin Scale.

To establish a mortality prediction model for elderly patients with CM, we conducted a multivariable logistic regression analysis on the four selected variables. The AUC of the predictive model was 0.81 (95% CI 0.71-0.91), and the internal validation using the bootstrap method (resampling = 1000) was 0.79 (95% CI 0.70-0.87) (Fig 5). To develop the predictive model, we constructed a nomogram, which provides us with a convenient and personalized tool for predicting the mortality in elderly CM patients (Fig 6).

The proposed model demonstrated well-calibrated (S3 Fig), with the Hosmer-Lemeshow test showing no statistical bias between the predicted and observed values (*P* = 0.82), suggesting a good model fit. DCA further demonstrated the clinical utility of the nomogram (S4 Fig), confirming its potential to benefit clinical decision-making in predicting mortality among elderly CM patients.

## Discussion

This study utilized the largest dataset of non-HIV CM cases in elderly patients to date, summarizing the clinical features, outcomes, and prognostic factors of this population. Elderly patients exhibited distinct clinical characteristics, including lower rates of typical symptoms like headache and vomiting but higher rates of altered consciousness and poor functional status. Notably, one-year all-cause mortality rate was significantly higher in elderly patients compared to non-elderly patients. We also developed the first pretreatment tool for identifying elderly patients at higher mortality risk, which could be crucial for guiding early and aggressive interventions.

Predisposing conditions were present in approximately one-third of cases across both age groups, with diabetes more prevalent among elderly patients. Diabetes is a well-established cause of immune dysfunction in the elderly and a known risk factor for infections, including CM [12]. Studies have shown that diabetic CM patients, regardless of HIV status, experience worse clinical outcomes and higher mortality rates, [13,14] likely due to impaired immune cell function and altered cytokine production [15].

Elderly patients were more likely to present with lower consciousness levels (GCS < 15) and greater functional disability (higher mRS scores) but reported fewer symptoms such as headache and vomiting, consistent with our previous findings [16]. Clinically, infectious diseases in the elderly may have presented with vague and elusive symptoms at onset, potentially mimicking cognitive decline or neurodegenerative diseases [17]. To avoid missed or delayed diagnoses, it is essential to conduct appropriate investigations for central nervous system (CNS) infections, especially in elderly patients presenting with altered consciousness. In elderly CM patients, the proportion of severe intracranial pressure elevation (> 300 mmH_2_O) was lower. Age may be an influencing factor, as previous study has reported that intracranial pressure in normal individuals also tends to decrease with increasing age [18]. This may explain why elderly patients experienced milder headache and vomiting.

CNS infections could cause the cerebral infarction, primarily related to infection-induced vascular damage and inflammatory responses [19,20]. Limited evidence suggests vasospasm and endarteritis may also play a role [21,22]. In our study, brain MRI revealed an incidence of acute infarction of 9.9% in elderly patients. Although this rate is lower than that reported in previous studies, [23,24] it remained significantly higher than in non-elderly patients. Elderly patients often have risk factors for cerebrovascular disease, such as diabetes and hypertension, leading to higher incidence rate of cerebral atherosclerosis and arterial stenosis. Clinically, acute ischemic stroke can sometimes be mistaken for cryptococcomas due to similar imaging features. The key difference is that ischemic infarcts typically show progression over time on follow-up MRI, whereas cryptococcomas exhibit persistent restricted diffusion. This distinction is due to ischemic infarcts being associated with transient ischemia-related cytotoxic edema, while cryptococcomas contain proteinaceous material that causes persistent diffusion abnormalities [25]. Cryptococcal basal ganglia lesions are believed to result from cryptococci entering the perivascular spaces along the penetrating arteries, which are part of the glymphatic circulation system. This can lead to the dilatation of VR spaces and, in severe cases, the formation of gelatinous pseudocysts [25]. In our study, the incidence of basal ganglia lesions was observed to be lower in elderly patients compared to non-elderly patients, possibly related to glymphatic circulation system impairment caused by arteriosclerosis in elderly patients, [26]. which may hinder cryptococcal invasion into the VR spaces.

The one-year all-cause mortality rate for elderly CM patients was 31.2%, significantly higher than the 13.8% observed in non-elderly patients (*P *< 0.001). Identifying mortality predictors specific to elderly patients is crucial for guiding proactive clinical management. The main advantage of our model was that it allows physicians to use early and simple indicators such as clinical scoring, laboratory tests, and imaging findings to predict mortality in elderly CM patients.

The mRS score, commonly used to evaluate functional disability, was strongly correlated with poor outcomes and increased mortality in elderly CM patients. This underscores the importance of incorporating mRS into comprehensive assessments. Elevated blood leukocyte counts were also linked to worse outcomes, likely reflecting heightened inflammatory responses [27,28]. Monitoring leukocyte counts could help identify high-risk patients early.

Although the *Cryptococcus* smear count method based on CSF India ink staining has drawbacks, such as result instability, subjectivity, and limited positive rates, it remains a simple, accessible, and inexpensive rapid diagnostic method [29,30]. The baseline smear count reflects the fungal burden and has been shown in previous studies to influence patient prognosis [31–33]. Our findings showed that elderly survivors had significantly lower initial *Cryptococcus* smear count compared to those who died. The initial *Cryptococcus* smear count, to some extent, reflects the fungal load, and it is a risk factor for mortality. Cryptococcal basal ganglia lesions reflect the process by which *Cryptococcus* enters the perivascular spaces of the brain parenchyma, leading to dilatation of VR spaces and the formation of gelatinous pseudocysts [25,34]. The dilation of VR spaces is often considered a marker of glymphatic waste clearance dysfunction [35,36]. These lesions are highly likely to affect the glymphatic system and impede the penetration of antifungal drugs, resulting in poor treatment outcomes, and become an independent mortality risk factor, especially in elderly patients.

The delayed diagnosis of CM occurred in both elderly and non-elderly patients, with median onset-to-diagnosis intervals exceeding three weeks (22 and 27 days, respectively). Such delays may reflect the atypical clinical presentation of CM in the non-HIV population, particularly among elderly individuals, which can obscure early recognition. These results underscore the urgent need for greater clinical awareness and timely diagnostic evaluation of suspected CNS infections in non-HIV patients to avoid missed or delayed diagnoses and improve patient outcomes.

Our study has several limitations. Although we applied robust statistical methods to adjust for confounding, its retrospective nature still makes it vulnerable to bias, which may affect generalizability. As a tertiary referral center for non-HIV CM, we manage both local cases and referrals from other hospitals, which may lead to selection bias. Furthermore, despite the large cohort size, which enhances the significance of the results, the single-center nature of the study may limit the applicability of our findings to other populations or healthcare settings. In addition, CrAg testing, a highly sensitive and specific diagnostic tool, was only introduced at our center in 2019 [37]. The limited data available in this study prevented us from evaluating its diagnostic utility in elderly patients and its potential predictive value. Prospective multicenter studies with standardized diagnostic protocols are needed to validate our findings and refine the predictive model.

In conclusion, this study provides new insights into the clinical features and mortality risk factors of CM in non-HIV elderly patients. The predictive model we developed has the potential to improve the management and outcomes of this vulnerable population. Future research should focus on validating this model in diverse patient populations and developing early interventions based on confirmed risk factors to reduce mortality.

## Supporting information

S1 Fig Analysis of factors contributing to the prediction model. (A) Univariate logistic regression identifies individual variables significantly associated with the outcome. (B) A heatmap displaying the pairwise Pearson correlation coefficients among all candidate predictive variables analyzed. The color gradient indicates correlation magnitude: darker red denotes stronger negative correlations, while blue shades represent stronger positive correlations. *P < 0.05; **P < 0.01; ***P < 0.001. ALT = alanine aminotransferase; AST = aspartate aminotransferase; AUC = area under the curve; CSF = cerebrospinal fluid; mRS = Modified Rankin Scale; WBC = white blood cells.
(TIF)

S2 Fig Predictor selection via LASSO regression analysis. (A) The tuning parameter (λ) was selected based on the deviance. The dotted lines represent the minimum criteria (left) and the 1-SE criteria (right). (B) A coefficient profile plot shows the evolution of the coefficients for each variable as log(λ) changes. In this study, the final selection of predictors was based on values between the minimum and 1-SE, resulting in four nonzero coefficients (mRS score, *Cryptococcus* smear count, leukocyte count, basal ganglia lesions). LASSO = Least Absolute Shrinkage and Selection Operator (LASSO); mRS = Modified Rankin Scale; SE = standard error criteria.
(TIF)

S3 Fig Calibration curves for the prediction model. The plot compares predicted (x-axis) versus observed (y-axis) one-year survival probabilities. The dashed line represents ideal calibration; the solid line (bias-corrected) shows slight overestimation of risk at higher probabilities. The Hosmer-Lemeshow test confirmed good fit (*P* = 0.82).
(TIF)

S4 Fig Decision curve analysis for assessing the clinical utility of the prediction model. The model showed clear net clinical benefit across a range of threshold probabilities compared to “treat-all” and “treat-none” strategies.
(TIF)
