# Supplementary figures and images for: Clinical features and mortality risk factors in non-HIV elderly patients with cryptococcal meningitis: A retrospective cohort study from 2013 to 2022
Source: PLoS Negl Trop Dis. 2025 Sep 11;19(9):e0013521. doi: 10.1371/journal.pntd.0013521 (PMC12445562; doi:10.1371/journal.pntd.0013521)

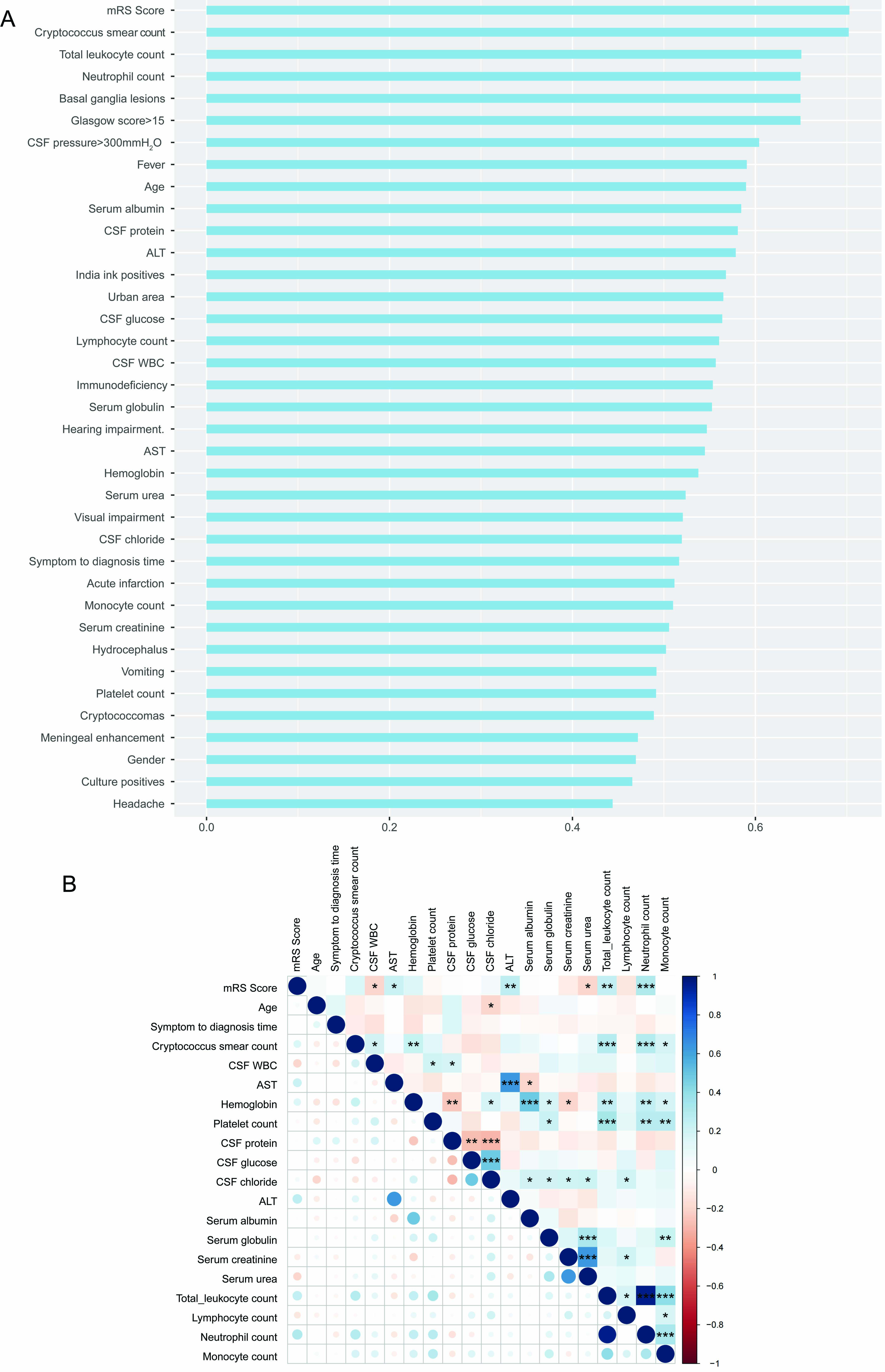

Supplement: S1 Fig — (A) Univariate logistic regression identifies individual variables significantly associated with the outcome. (B) A heatmap displaying the pairwise Pearson correlation coefficients among all candidate predictive variables analyzed. The color gradient indicates correlation magnitude: darker red denotes stronger negative correlations, while blue shades represent stronger positive correlations. *P < 0.05; **P < 0.01; ***P < 0.001. ALT = alanine aminotransferase; AST = aspartate aminotransferase; AUC = area under the curve; CSF = cerebrospinal fluid; mRS = Modified Rankin Scale; WBC = white blood cells. (TIF) [file pntd.0013521.s001.tif]

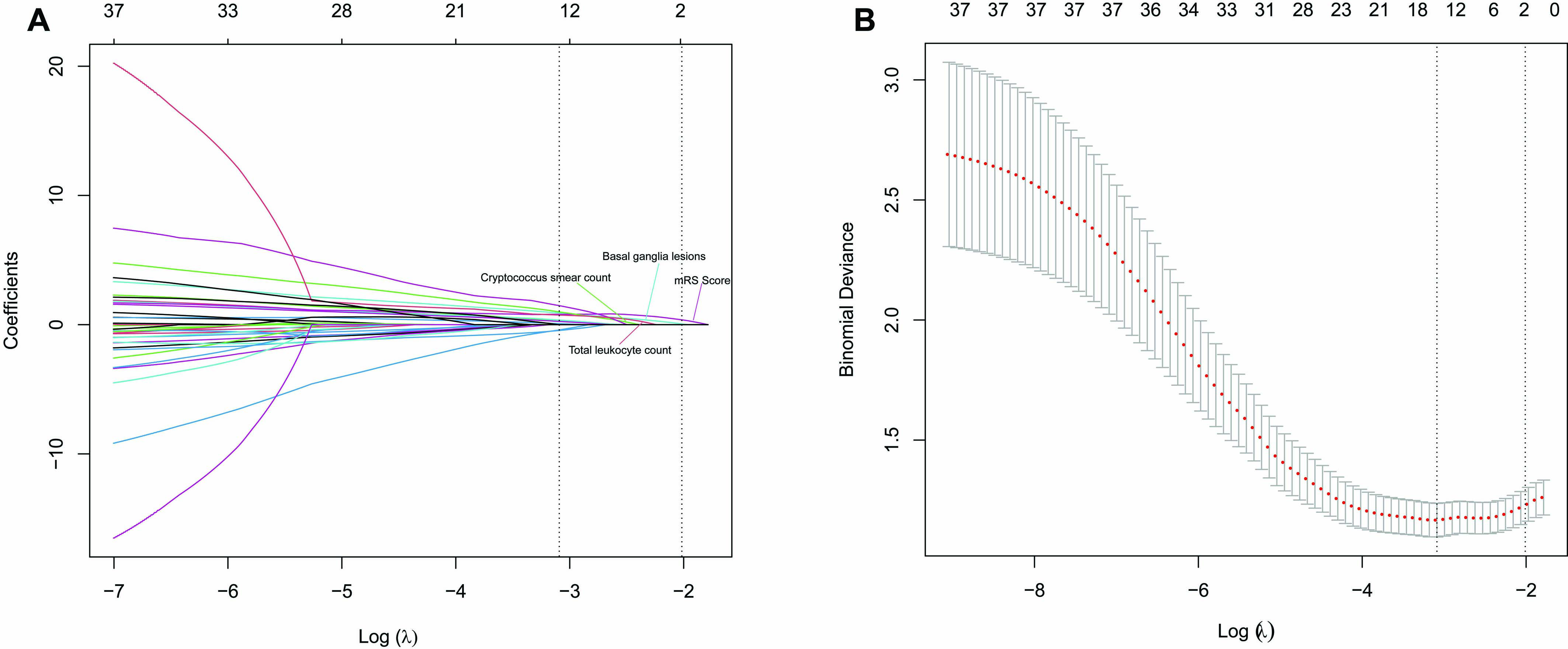

Supplement: S2 Fig — (A) The tuning parameter (λ) was selected based on the deviance. The dotted lines represent the minimum criteria (left) and the 1-SE criteria (right). (B) A coefficient profile plot shows the evolution of the coefficients for each variable as log(λ) changes. In this study, the final selection of predictors was based on values between the minimum and 1-SE, resulting in four nonzero coefficients (mRS score, Cryptococcus smear count, leukocyte count, basal ganglia lesions). LASSO = Least Absolute Shrinkage and Selection Operator (LASSO); mRS = Modified Rankin Scale; SE = standard error criteria. (TIF) [file pntd.0013521.s002.tif]

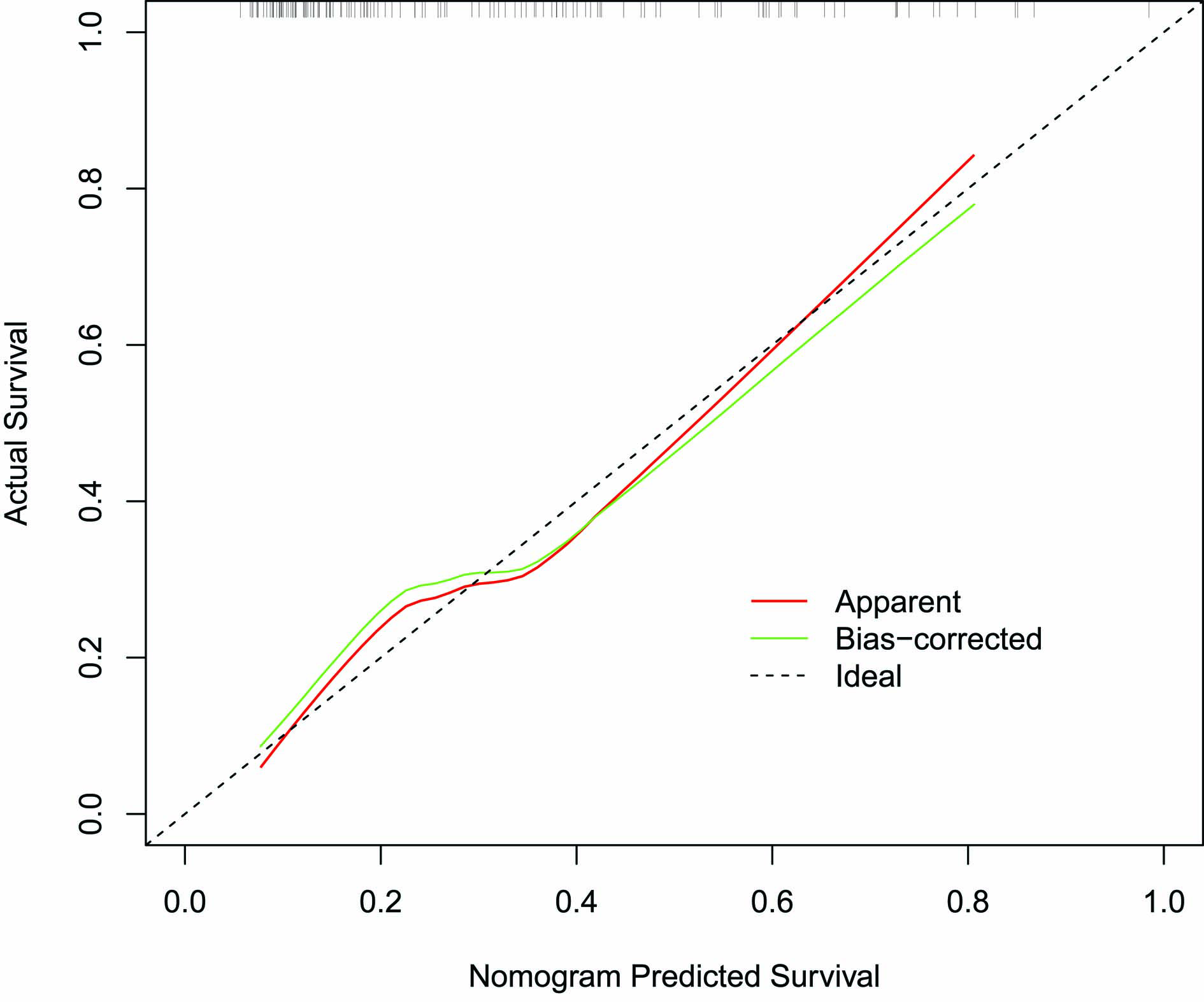

Supplement: S3 Fig — The plot compares predicted (x-axis) versus observed (y-axis) one-year survival probabilities. The dashed line represents ideal calibration; the solid line (bias-corrected) shows slight overestimation of risk at higher probabilities. The Hosmer-Lemeshow test confirmed good fit (P = 0.82). (TIF) [file pntd.0013521.s003.tif]

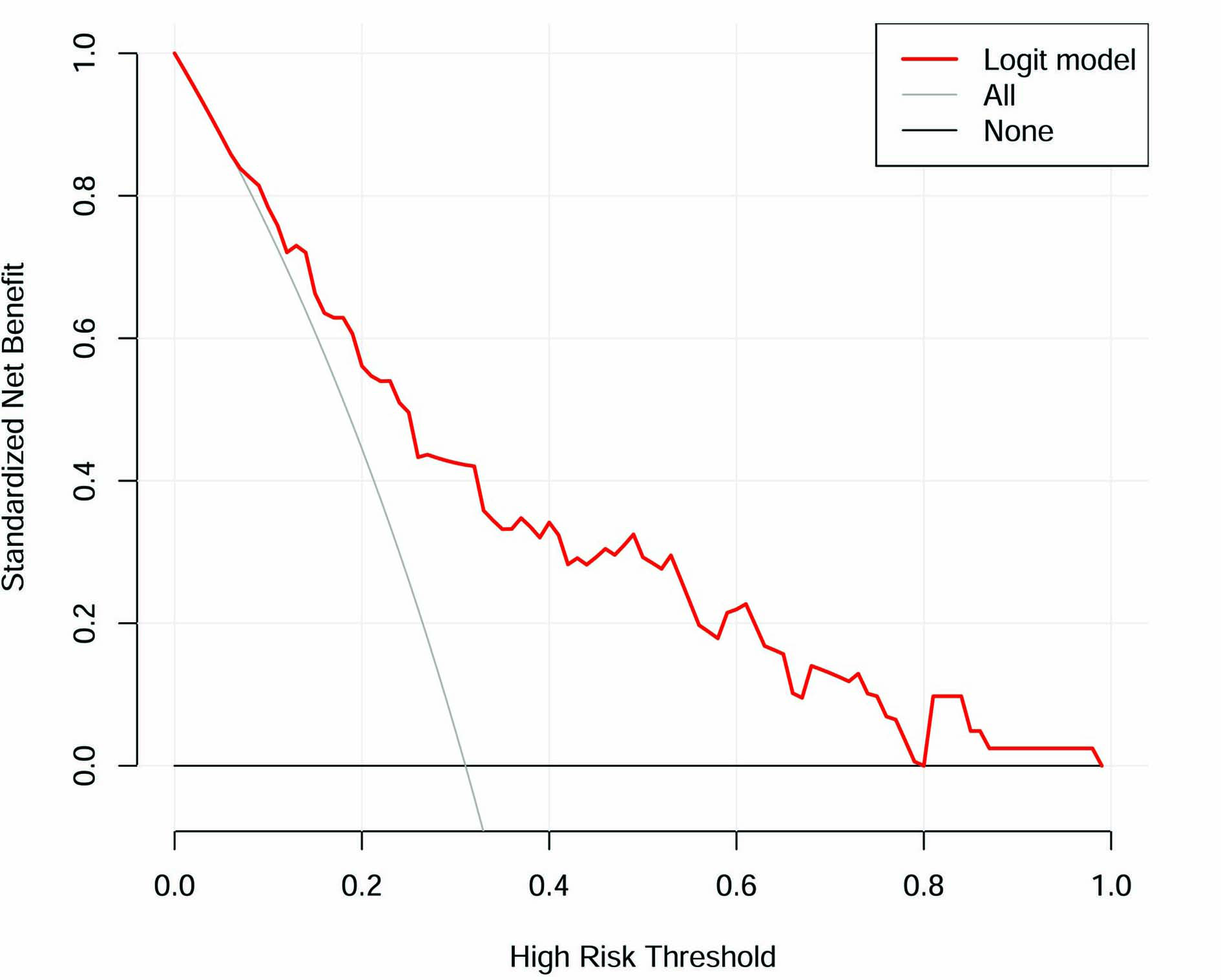

Supplement: S4 Fig — The model showed clear net clinical benefit across a range of threshold probabilities compared to “treat-all” and “treat-none” strategies. (TIF) [file pntd.0013521.s004.tif]
